# Common reasons for not accepting manuscripts for further processing after editor’s triage and initial screening

**DOI:** 10.12669/pjms.35.1.28

**Published:** 2019

**Authors:** Shaukat Ali Jawaid, Masood Jawaid

**Affiliations:** 1 Shaukat Ali Jawaid Chief Editor, Pakistan Journal of Medical Sciences, Karachi - Pakistan; 2 Masood Jawaid Associate Editor, Pakistan Journal of Medical Sciences, Karachi - Pakistan

One of the most common complaints by the authors against the editors is their failure to update them about the developments regarding their submitted manuscripts. It is alleged that some editors keep sitting on the manuscripts for weeks and months without providing any initial feedback. We in the Pakistan Journal of Medical Sciences most often check all the new submissions within two weeks, at times may be within ten days and authors of those manuscripts which are not accepted for further processing due to various reasons after initial screening and editor’s triage, are informed accordingly. We take the decision after reading the abstract most often and after going through the full manuscript in a few selected cases. They are also conveyed the reasons for such a decision.

The percentage of such manuscripts which are not processed further varies from 70-80% which may look rather high but since we have too many submissions as compared to the number we can handle due to human resource and financial constraints, we believe it is better to give feedback the authors as soon as possible so that they can submit their manuscripts to some other journal. Editors have given these process different names i.e. desk rejection, sudden death rejection, immediate rejection, initial triage and editor’s triage. Publishing a quality peer reviewed journal in limited resource countries is a very stressful and frustrating job.^1^,^2^ The editors are faced with many problems and authors are the most dangerous pressure group which they have to face.^3^ Authors are very impatient and they wish to see whatever they have written in print immediately and to achieve this objective they put all sort of pressures on the editors. Many times it is author’s failure to carefully read and follow the instructions to authors of the journal to which they are submitting the manuscripts for publication which increases the trauma to their manuscripts.^4^ Of course it is quite painful for the authors to hear about the rejection of their manuscripts but the fault does not lie with the editors all the time.

The percentage of rejection after initial screening or editor’s triage will vary from journal to journal keeping in view their human, financial resources and other facilities.^5^ Some well-established journals which have lot of staff, look at all new submissions within two to three days and a decision of desk rejection is conveyed to the authors immediately. We believe that it is much better to use the phrase *“not accepted for further processing”* rather than desk rejection because many a times the papers not accepted for external peer review are of very good quality but since the journal cannot handle all the submissions, they are rejected.

More recently this topic of desk rejection was discussed in detail on the WAME list serve.^6^ The discussion was initiated by Mary Ellen Kerans from Spain who put forward three questions and sought opinion from other editor colleagues. The three very pertinent questions asked were:

1. Has the desk rejection increased?
2. How long a manuscript does stays with the journal before a letter of desk rejection is generated?
3. What process is involved in desk rejection?

During the discussion it was also pointed out that it is the amazing number of authors who do not read the aims and scope of the journal, instructions for authors before making submission. Editors have no other option but to reject those papers with numerous flaws in methodology, some would filter quality of writing. Some papers require complete re-write to improve English language and Grammar. Many manuscripts require proper copy edit before these papers are sent for external review but many journals may not have this facility. Journals with high rate of submissions, high impact factor have highest rate of rejection after initial screening and this desk rejection rate is increasing. Moving from e mail to online submissions direct on the journal website has on one hand significantly increased the submissions but it has also increased rejection after initial screening as the manuscripts are not found suitable for the journal for various reasons.

Some use the term “Primary Editorial Rejection” but the process is the same. In Pakistan Journal of Medical Sciences, final decisions in all such cases of rejection after initial screening is taken by the Chief Editor or another senior editor, section editors who have been entrusted the responsibility of further handling of the manuscripts. Some of the most common reasons for this rejection without external peer review are as under:

1. Author’s failure to follow instructions on the journal website.
2. Too lengthy manuscripts with too many tables, illustrations.
3. Poor English and Grammar.
4. Failure to upload proper Letter of Undertaking signed by all listed authors regarding exclusive submissions.
5. Too many authors with strong suspicion of gift authorship.
6. Already too many papers in the same specialty are under process and pending.
7. Paper being more suitable for a specialty journal rather than a general medical journal.
8. Papers dealing with local problems which are more suitable for local publication by the authors in their own country.
9. Not falling within the scope of the journal.
10. Poor literature search, very old references.
11. Too many submissions than the journal can handle due to human and financial resource constraints.
12. Clinical trials which are not registered.
13. Failure to provide Ethics Committee, Institutional Board approval of the study protocol.
14. Editor’s bias also plays a role. They prefer those manuscripts on topics which are of interest to them. For example in Pakistan Journal of Medial Sciences, we prefer manuscripts like RCTs, epidemiological studies, innovative surgical procedures, innovations in medical education, medical ethics, patient safety and so on while KAP studies, survey reports, case reports, narrative reviews have a very low priority. Systematic Reviews and Meta-Analysis are also preferred provided the topic is interesting and they are not too lengthy.
15. Previous history of the submitting author. Some refuse to learn from their previous mistakes, withdrawal after getting their papers peer reviewed and then submitting it to some other journal, failure to pay publication charges and asking for waivers after acceptance of manuscripts though they are supposed to make such requests at the time of submission.
16. It also depends on the number of manuscripts a journal includes in an issue. In case of online only journals, they can include a very large number of manuscripts but for those journals that have to produce print issue as well in addition to online issue, it is very expensive.
17. Regulatory bodies in many countries do not give importance and same weightage to manuscripts published in “only Online Journals”, hence the editors have no other option but to keep the number of manuscripts accepted for further processing and publication limited to a number which they can easily handle and manage.

A study by Meyer HS et al published in Academic Medicine in 2018 had highlighted the following reasons for immediate rejection of papers after initial screening: 7

1. Ineffective study question and/or design;
2. Suboptimal data collection process;
3. Weak discussion and/or conclusions;
4. Unimportant or irrelevant topic to the journal’s mission;
5. Weak data analysis and/or presentation of results;
6. Text difficult to follow, to understand;
7. Inadequate or incomplete introduction;
8. Other publishing considerations;
9. Issues with scientific conduct.

Manuscripts had, on average, three or more reasons for rejection. These reasons are quite similar to our own findings as well as the points raised by other distinguished editors in the discussion on WAME list serve.

Writing in an editorial, Garg A et al from Journal of Clinical and Diagnostic Research reported that they did retrospective analysis of one thousand manuscripts submitted to their journal since August 1^st^ 2014. Of the 902 papers which reached the end point on decision were considered. They rejected 552 out of this 902 papers after initial screening. The most common reasons for this decision were commonality of subjects, non-compliance by the authors, flaws in methodology. Other reasons they listed included plagiarism, poor drafting, having published on same topic, inconsistency in data, mismanagement, black listed authors, ethical issues and out of scope of the journal.^8^ Here again the reasons are similar to our findings as well as the points raised by other editors.

## Fast Track Processing

If the topic is interesting and there are chances of more citations, we do ask the authors to rectify the deficiencies and resubmit the manuscript. The facility of fast track processing is offered in a few selected cases just to help those who have either to appear in Fellowship Exam of CPSP or defend their PhD Thesis. Even in those cases the authors have to make a request, justify it and convince the editor. If their request for fast track processing is accepted, they get peer review report within ten weeks after complete submission but there is no guarantee of acceptance and publication. Most often we do not accept requests for fast track processing unless we have looked at the manuscript because, we have noted that many authors are under the false impression that by paying fast track processing fee, they can increase the chances of acceptance of their manuscripts. This is not true and the decision on acceptance is purely based on the quality of the manuscript and not the author’s ability to pay.

Yet another problem that the editors in Pakistan face is the decision by Higher Education Commission as well as Pakistan Medial & Dental Council besides medical institutions which prefer publication of manuscripts in Impact Factor Journals. In Pakistan we have just three biomedical journals i.e. Pakistan Journal of Medical Sciences, Journal of Pakistan Medical Association and Journal of College of Physicians & Surgeons Pakistan which are covered by Clarivate Analytics USA Web of Sciences and enjoy Impact Factor. Hence, they are under too much pressure. The solution lies in improving the standard of the journals and getting more and more Pakistani biomedical journals in the Web of Science to earn Impact Factor. Editors of quite a few medical journals in Pakistan are currently striving hard to earn this and hopefully in the coming few years, more Pakistani medical journals might get covered by Web of Sciences. HEC as well as PM&DC are also taking initiatives to help the journals through different ways including arranging training courses but still a lot more needs to be done. One of the important steps which institutions like the HEC needs to take is provision of facility of generating XML files for submission on PubMed Central. This will ensure that more and more Pakistani medical journals are visible on the PubMed. Increased visibility will lead to increased citations which will go a long way in increasing our contributions to the world medical literature.
